# Multiply Resistant (MR) *Salmonella enterica* Serotype Typhimurium DT 12 and DT 120: A Case of MR DT 104 in Disguise?

**DOI:** 10.3201/eid0804.010348

**Published:** 2002-04

**Authors:** Andrew J. Lawson, Miatta U. Dassama, Linda R. Ward, E. John Threlfall

**Affiliations:** Central Public Health Laboratory, London, United Kingdom

**Keywords:** Salmonella Typhimurium, MR DT 104, antibiotic resistance, molecular subtyping

## Abstract

Multiresistant *Salmonella enterica* serotype Typhimurium definitive phage type (DT) 12 and DT 120 are more closely related to DT 104 than to non-multiresistant strains of their respective phage types. Multiresistant DT 12 and DT 120 appear to have arisen due to changes in phage susceptibility of DT 104 rather than horizontal transfer of resistance genes.

Multiresistant (MR) *Salmonella enterica* serovar Typhimurium definitive phage type (DT) 104 is now acknowledged as an internationally distributed zoonotic pathogen. Since 1991, this phage type has been second only to *S.* Enteritidis phage type 4 as the principal agent of human salmonellosis in England and Wales (1). MR DT 104 is characterized by resistance to ampicillin, chloramphenicol, streptomycin, sulfonamides, and tetracyclines (R-type ACSSuT). In MR DT 104 these antibiotic resistance genes have been accumulated in chromosomally encoded gene cassettes, a process mediated by the presence of class 1 integrons (2). Some isolates possess additional plasmid-mediated resistance to trimethoprim and low-level resistance to ciprofloxacin because of point mutations in the *gyr*A gene (1).

The potential exists for the horizontal transfer of genetic elements such as antibiotic resistance gene cassettes between *Salmonella* serotypes and phage types. Evidence of this transfer was reported in 2000, when the presence of an MR DT 104-like antibiotic resistance gene cluster was reported in *S.* Agona (3). Throughout the 1990s, the ACSSuT R-type was also identified in isolates of *S.* Typhimurium DT 12 and DT 120, although as yet numbers remain relatively small (27 of 84 and 22 of 109 of DT 12 and DT 120 isolates, respectively, were of R-type ACSSuT of a total of 2,651 *S.* Typhimurium isolates received at the Central Public Health Laboratory, London, in 2000). ACSSuT-resistant MR DT 12 incidence has remained fairly constant since the mid-1990s, but as isolations of sensitive DT 12 have diminished the relative proportion of MR DT 12 has increased (Figure 1). ACSSuT-resistant DT 120 has attained levels comparable with those of MR DT 12 only in the last few years but might well have been the predominant representative of DT 120, were it not for a large outbreak of antimicrobial-sensitive DT 120 in northern England during 1999-2000 (Figure 1) (4).

**Figure 1:**
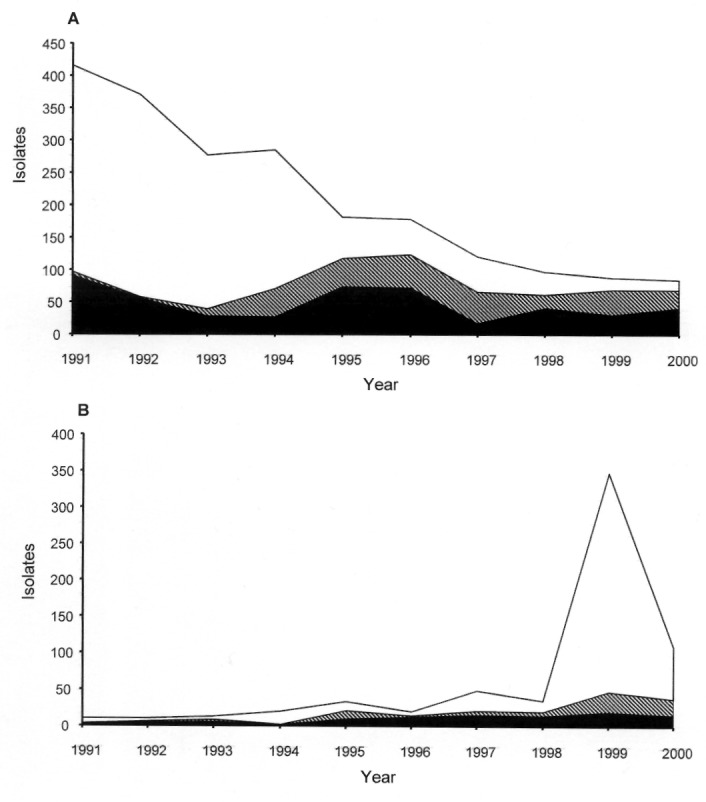
Antimicrobial susceptibility of *Salmonella enterica* serotype Typhimurium definitive phage type (DT) 12 and DT 120 isolates, England and Wales, 1991–2000. A, *S.* Typhimurium DT 12; B, *S.* Typhimurium DT 120. Clear bar, sensitive; diagonal screened bar, resistance to ampicillin, chloramphenicol, streptomycin, sulfonamides, and tetracyclines (ACSSuT; includes resistant-type ACSSuT and ACSSuT plus additional resistances to Tm, Cp_L,_ or both); black bar, other resistance patterns**.**

We investigated multiple antibiotic-resistant strains of MR DT 12 and MR DT 120 to determine if horizontal transfer of the antibiotic resistance genes had occurred between apparently unrelated phage types of *S.* Typhimurium.

## The Study

Isolates of *S.* Typhimurium DT 12 and DT 120 from human, animal, food, and environmental sources received by the Laboratory of Enteric Pathogens from 1991 to 2000 were examined. For each phage type, 40 strains were selected, consisting of 15 antibiotic-sensitive strains, 12 strains of R-type ACSSuT, and 13 strains with non-ACSSuT R-types. Control strains of *S.* Typhimurium DT 104, both sensitive and MR, were also included.

All strains were characterized by phage type, in accordance with the scheme of Anderson et al. (5); antimicrobial susceptibility patterns were determined by using the breakpoint method (6), so that final concentrations (mg/mL^-1^) were ampicillin, 8; chloramphenicol, 8; furazolidone (Fu), 8; gentamicin (G), 4; kanamycin (K), 8; neomycin (Ne), 8; streptomycin, 16; sulfonamides, 64; tetracyclines, 8; trimethoprim, 2; and ciprofloxacin, 0.125.

The presence of the 13 kbp ACSSuT resistance gene cluster was determined by a long-polymerase chain reaction (PCR) method developed by Walker et al. (7) that amplified the 10- kbp region between the *aad*A2 (streptomycin resistance) and *bla*_CARB-2_ (ampicillin resistance) genes on the sequence of the antibiotic resistance gene cluster from *S.* Typhimurium DT 104 isolate H3380 (8). All strains of MR DT 104, DT 12, and DT 120 of R-type ACSSuT produced 10-kbp PCR amplicons; strains with other resistance patterns were negative.

The strains were examined for the presence of class 1 integrons by using a specific PCR assay (9). All strains of MR DT 104, DT 12, and DT 120 of R-type ACSSuT produced bands of 1.0 kbp and 1.2 kbp corresponding to the two integrons reported within the resistance gene cassettes (8,10). This corroborated the long-PCR result and demonstrated that there was no apparent variation in the arrangement of the genes within the ACSSuT resistance cassettes. Sensitive and non-ACSSuT-resistant strains were either negative by the class 1 integron PCR or produced bands other than 1.0 kbp and 1.2 kbp.

**Figure 2:**
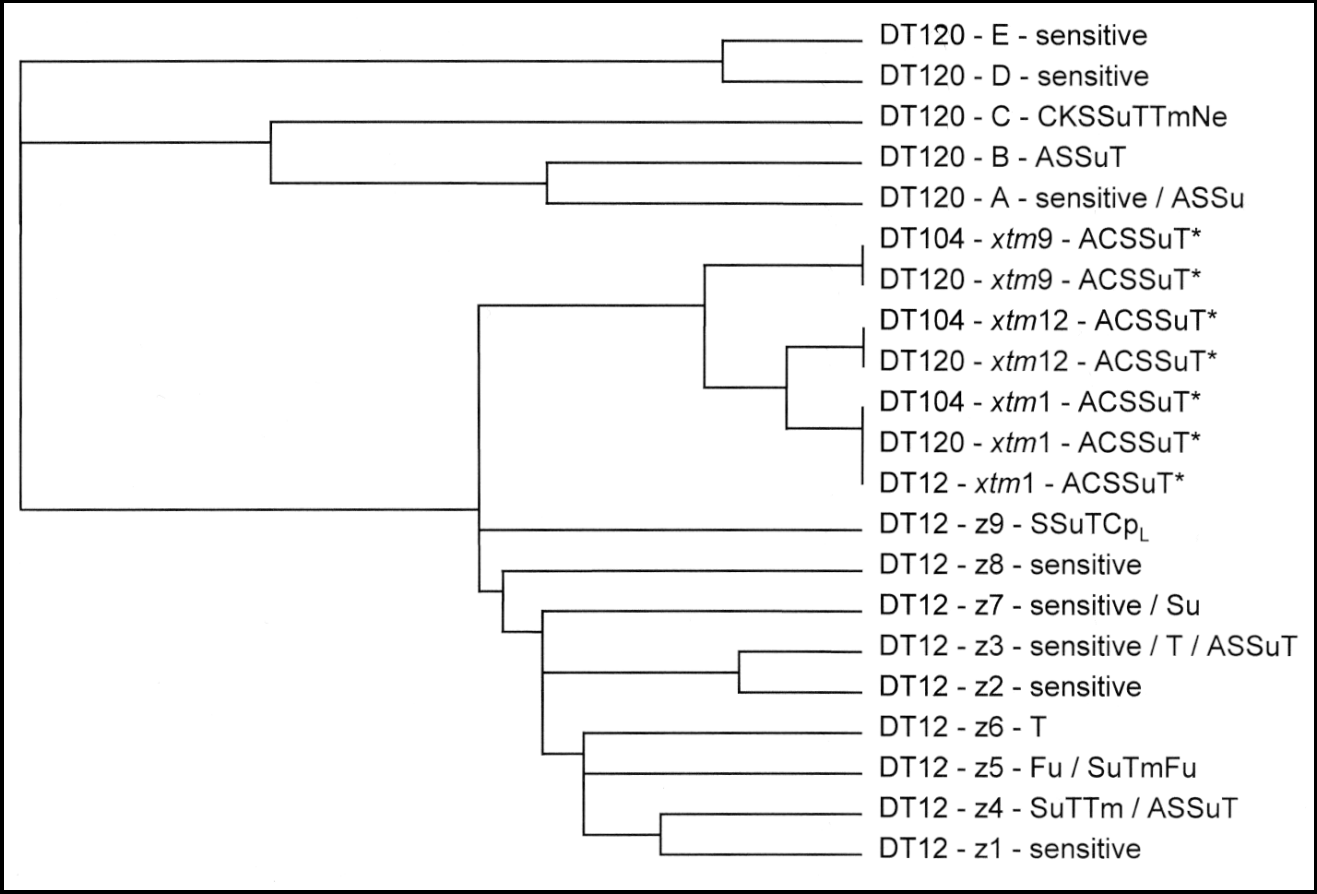
Dendrogram showing the relationships of pulsed-field get electrophoresis profiles by the nearest neighbor technique.*Includes resistant (R)-type ACSSuT and ACSSuT plus additional resistances to Tm, Cp_L,_ or both. A = ampicillin, C = chloramphenicol, Fu = furazolidone, K = kanamycin, Ne = neomycin, S = streptomycin, Su = sulfonamides, T = tetracyclines, Tm = trimethoprim, Cp_L_ = ciprofloxacin.

Pulsed-field gel electrophoresis (PFGE) analysis was performed by using the restriction enzyme *Xba*I according to the method of Powell et al. (11). Analysis showed that all sensitive and non-ACSSuT-resistant strains of DT 12 and DT 120 examined formed distinct groups of phage type-related PFGE profiles that clustered when analyzed by the nearest neighbor algorithm (12) (Figure 2). Nine distinct profiles (z1 to z9) were obtained from non-ACSSuT-resistant DT 12 and five different profiles (A to E) from the non-ACSSuT-resistant DT 120. Importantly, the ACSSuT-resistant strains of both DT 12 and DT 120 produced similar PFGE patterns that were distinct from those obtained by non-ACSSuT-resistant strains of the same phage type. However, these patterns were identical to those commonly observed in both sensitive and MR strains of DT 104 (profiles *xtm*1, *xtm*9, and *xtm*12). In contrast, the PFGE profiles of sensitive and ACSSuT-resistant strains of DT 104 typically differ by no more than a few bands. The PFGE profile of the ACSSuT-resistant DT 12 strains were all identical to that produced by most (80%- 90%) DT 104 isolates, termed profile *xtm*1 (10). The ACSSuT-resistant strains of DT 120 were mainly (8 of 12 strains) profile *xtm*1; the remainder were profiles *xtm*12 (3 of 12) and *xtm*9 (1 of 12). PFGE profiles *xtm*9 and *xtm*12 are less common variants of the basic *xtm* 1 pattern in DT 104. These variants occur in <5% of isolates and differ from the predominant pattern by two bands and one band, respectively (A. J. Lawson, unpub. data) (Figure 2).

Strains were analyzed for presence of plasmids by standard methods (13). All DT 104 and DT 12 strains contained a 90-kbp “serotype-specific” plasmid commonly found in *S.* Typhimurium (14). However, in DT 120 only those strains of R-type ACSSuT possessed this plasmid. Purified 90-kbp plasmid extracts from R-type ACSSuT strains of DT12, DT120, and DT104 were negative for integron-associated resistance gene cassettes by PCR (as described above), confirming the chromosomal location of the antibiotic resistance genes.

## Results

Strains of *S.* Typhimurium DT 12 and DT 120 of R-type ACSSuT possess antibiotic resistance gene cassettes indistinguishable from those typically present in multiresistant strains of the internationally distributed zoonotic pathogen *S.* Typhimurium DT 104. Strains of MR DT 12 and MR DT 120 of R-type ACSSuT have PFGE profiles distinct from those of other strains of the same phage type. Furthermore, the PFGE profiles of ACSSuT-resistant MR DT 12 and MR DT 120 are identical to those previously reported in MR DT 104. Additionally, ACSSuT-resistant strains of MR DT 120 possess a 90-kbp plasmid absent in DT 120 strains of other R-types, but present in DT 12 and DT 104 strains.

These data suggest that the occurrence of R-type ACSSuT in *S*. Typhimurium DT 12 and DT 120 strains is probably due to changes in phage susceptibility in a small proportion of MR DT 104 strains rather than to horizontal transfer of a resistance gene cassette. The Table shows selected phage reactions used in the *S*. Typhimurium phage typing scheme (5) and illustrates the similarity between the phage susceptibility profile of DT 12 and DT 120 and that of DT 104, especially the weaker reacting 104L profile. The causes of changes in phage susceptibility are complex and difficult to trace but are thought to result from the acquisition of new phages or plasmids and to changes in bacterial cell surface phage-receptors.

**Table:** *Salmonella enteric* serotype Typhimurium phage typing scheme, selected phage reactions

Phage type	Selected routine phages																Additional phages				
	8	10	11	12	13	14	15	16	17	18	20	27	28	29	32	35	1	2	3	10	18
104	-/±	-	-	CL	CL	-	-	-	-	CL	+/±	+/±	-/±	-	-/±	+/±	-	-	-	OL	-
104L	-/±	-	-	++	++	-	-	-	-	+/SCL	-	-	-	-	-	-	-	-	-	OL	-
12	-	-	-	CL	CL	-	-	-	-	-	-	-	-	-	-	-	SCL	SCL	SCL	OL	-
12L	-/±	-	-	CL	CL	-	-	-	-	-	-	-	-	-	-	-	-	-	-	OL	-
120	-	-	-	-	-	-	-	-	-	CL	-	-	-	-	-	-	red	red	red	OL	-

L = light or reduced reaction with typing phages; CL = clear lysis; OL = opaque lysis; SCL = semi-confluent lysis; red = reduced zone; ±, +, ++ = increasing numbers of discrete plaques, - = no reaction.

With regard to the international distribution of these strains, the ACSSuT-resistant DT 12 strains were all PFGE profile *xtm*1, and all originated from patients in England and Wales with no history of foreign travel. In contrast, the ACSSuT-resistant DT 120 strains consisted of a mixture of *xtm*1, *xtm*9, and *xtm*12 PFGE profiles and included cases from South Africa (*xtm*1), Sweden (*xtm*12), Northern Ireland (*xtm*9), and an isolate from a patient with a history of travel to the West Indies (*xtm*1). Continued surveillance coupled with molecular typing should be maintained to follow the spread of these new MR DT 104 variants in animals and humans.
